# When "no" might not quite mean "no"; the importance of informed and meaningful non-consent: results from a survey of individuals refusing participation in a health-related research project

**DOI:** 10.1186/1472-6963-7-59

**Published:** 2007-04-26

**Authors:** Brian Williams, Linda Irvine, Alison R McGinnis, Marion ET McMurdo, Iain K Crombie

**Affiliations:** 1 Section of Public Health, Division of Community Health Sciences, Mackenzie Building, Kirsty Semple Way, University of Dundee, Dundee, UK; 2 Division of Medicine & Therapeutics, Ninewells Hospital & Medical School, University of Dundee, Dundee, UK

## Abstract

**Background:**

Low participation rates can lead to sampling bias, delays in completion and increased costs. Strategies to improve participation rates should address reasons for non-participation. However, most empirical research has focused on participants' motives rather than the reasons why non-participants refuse to take part. In this study we investigated the reasons why older people choose not to participate in a research project.

**Methods:**

Follow-up study of people living in Tayside, Scotland who had opted-out of a cross-sectional survey on activities in retirement. Eight hundred and eighty seven people aged 65–84 years were invited to take part in a home-based cross-sectional survey. Of these, 471 refused to take part. Permission was obtained to follow-up 417 of the refusers. Demographic characteristics of people who refused to take part and the reasons they gave for not taking part were collected.

**Results:**

54% of those invited to take part in the original cross-sectional survey refused to do so. However, 61% of these individuals went on to participate in the follow-up study and provided reasons for their original refusal. For the vast majority of people initial non-participation did not reflect an objection to participating in research in principle but frequently stemmed from barriers or misunderstandings about the nature or process of the project itself. Only 28% indicated that they were "not interested in research". The meaningfulness of expressions of non-consent may therefore be called into question. Hierarchical log-linear modelling showed that refusal was independently influenced by age, gender and social class. However, this response pattern was different for the follow-up study in which reasons for non-participation in the first survey were sought. This difference in pattern and response rates supports the likely importance of recruitment issues that are research and context specific.

**Conclusion:**

An expression of non-consent does not necessarily mean that a fully informed evaluation of the pros and cons of participation and non-participation has taken place. The meaningfulness of expressions of non-consent may therefore be a cause for concern and should be subject to further research. Many reasons for non-participation may be specific to a particular research topic or population. Information sheets should reflect this by going beyond standardised guidelines for their design and instead proactively seek out and address areas of concern or potential misunderstanding. The use of established behavioural theory in their design could also be considered.

## Background

Recent years have seen changes to consent procedures, data access, and research governance in the UK, all of which are designed to ensure that health research is conducted ethically and with integrity. The motivation for these changes are rooted in an increasing culture of accountability of professionals and a desire to address growing public and media concern about the ethical conduct of research. However, some of these developments may threaten the delivery of valid and reliable research [1,2]. At the centre of these challenges is the ongoing struggle to achieve high recruitment/response rates in community based research projects.

Low response and participation rates undermine statistical power and increase the probability of bias. Non-responders have, for example, been found to be more likely to be smokers, to have poorer health and to make less use of health care services [3-5]. Furthermore, these problems are exacerbated among particular social groups: response rates usually fall with age [6-8] and are lower in those with less education and poorer socio-economic status [9,10]. While knowing the magnitude of these differences may enable researchers to estimate or to adjust for the bias introduced by non-response [3,5,7], this is second best to a policy of prevention or reduction of initial non-response.

Numerous attempts have been made to identify effective strategies to maximise participation in both experimental and observational studies [11-13]. While a recent Cochrane review that identified 372 randomised controlled trials of interventions aimed to increase response rates to postal questionnaires identified a range of effective strategies [14] (envelope size, paper colour, financial incentives and ease of opt out) meta-analyses frequently show significant levels of heterogeneity between studies [15]. This indicates that strategies that may be highly effective for one social group in one setting may be less effective or even counterproductive in another. While this suggests that the reasons behind non-response or poor recruitment are likely to be variable, interventions to improve recruitment have seldom been based on the findings of exploratory studies of the *reasons *for non-response or recruitment in the first place. Consequently, even interventions that have some degree of demonstrable effectiveness are unlikely to be able to address issues that stem from the unique and idiosyncratic nature and context of individual projects where potential respondents may face specific practical problems or concerns.

Even where effectiveness is demonstrated questions remain. The finding that a particular strategy improves recruitment or response does not mean that it has either addressed the underlying concerns or practical problems associated with low rates. Instead it may simply be masking the problem. For example, monetary and non-monetary incentives may encourage people to participate but do nothing to alleviate their concerns about how the data are used or whether it will be treated confidentially.

Numerous studies have examined the motives and experiences of individuals who *participate *in research [16-21]. However, few have explored reasons for *non-participation*. This is unsurprising given the potential ethical and practical difficulties of accessing individuals who have already said that they do not wish to participate in a piece of research. However, structural, ethical and procedural changes for research (the increasing requirement to seek consent even where there is no patient contact, to have a formal "opt-in" rather than an "opt-out" process, and the increasing inability of health researchers in the UK to recruit participants directly rather than through a clinical intermediary) all mean that response rates have *increasing *potential for bias [22,23]. Consequently, it is perhaps more important than ever to focus not only on what motivates response and participation but rather what *non-response *or refusal to participate actually means and what the reasons behind it are. In this paper we report the findings of a survey of the characteristics and reasons for *non-response *in a study of attitudes to physical activity among older people. We then go on to discuss in detail possible responses and considerations. We do not present solutions but simply intend to encourage researchers to look at the issue of research non-participation as a behaviour that can be explored, analysed, and theorised just as other behaviours are similarly studied in other contexts of health.

## Methods

In 2002–4 we conducted a cross-sectional survey based on structured face to face interviews examining reasons for low physical activity among older people. These details, along with the findings of the study, have been reported elsewhere [24]. Subjects aged 65 to 84 years were identified from 16 GP practices in Dundee through age/gender registers. All were allocated a deprivation score [25]: scores of 1 and 2 were classed as areas of low deprivation; 3, 4 and 5 as medium; and 6 and 7 as areas of high deprivation [26]. A stratified random sample (age in 10 year groups, gender and three deprivation groups) of 1064 subjects was drawn. GPs screened the lists of names and 100 were excluded (terminal illness, dementia, living in a nursing home). Twenty subjects could not be traced at the addresses given. A further 57 subjects were excluded at initial contact because they were unsuitable (e.g. admitted to hospital, language barrier, bereavement, extreme frailty). A total of 887 subjects suitable for inclusion were invited to take part in the study.

Potential participants were initially contacted by a letter from their GP. This letter, and the information leaflet, stated that the study would investigate physical and social activities with the aim of improving facilities for older people. They were given the opportunity to opt out of the research by returning a postcard to the practice. If the postcard was not returned to the practice within three weeks, they were contacted by a research nurse to arrange an interview.

### Follow up of those who refused to be interviewed

We obtained permission from our Local Research Ethics Committee (LREC) for GPs to approach people who refused to take part in the main survey. We were aware that the our LREC, along with many others in the UK, were increasingly requiring researchers to use an "opt-in" approach in which individuals are written to by their general practitioner and asked to respond if they would like to receive more information about the project (as opposed to an "opt-out" method in which *non-response *to such a communication would result in the researchers approaching them). For the main survey the LREC originally requested an *opt-in *approach. However, after the researchers highlighted the degree to which sampling bias might be created in using an opt-in approach, the LREC agreed to the use of an opt-out approach. Given the preference for an opt-in stance and its underlying rationale to minimise burden on potential participants we were aware that any subsequent request to the LREC to approach people who had *already requested not to be involved *by actively telephoning or returning a card would be contentious. We therefore requested and obtained permission to ask only a small number of essential questions. We also agreed to ensure that the time required to complete the questionnaire be short, and that our covering letter overtly stress that participation was voluntary and that we respected their decision not to take part. These requirements precluded the possibility of using a qualitative approach to data collection. A further reason for the method and its brevity was simply to encourage as high a response rate as possible as it was assumed that people who had refused to take part in the main study would not be greatly motivated.

GPs sent a short questionnaire to those individuals who refused to take part in order to ascertain their reasons for non-participation. They were invited to return the questionnaire to their GP in a stamped addressed envelope which was provided. Only one letter was sent in order to minimize the potential intrusiveness of the study.

### The follow-up questionnaire

The follow-up questionnaire was designed to be as brief as possible in order to maximize likely response. Eight questions were selected by drawing on both the literature concerning non-response and poor recruitment, and the reasons expressed for non-response among those who had refused consent when the researcher had contacted them by telephone. The questionnaire included two questions on each of four topic areas: understandings about the research; concerns around privacy; personal reasons (e.g. health); lack of interest in research. A single open question was also asked in order to allow expression of other reasons not embodied in the previous eight.

### Analysis

Chi-squared tests were performed to investigate univariate associates with refusal. Model fitting using hierarchical log-linear modelling was used to identify significant independent predictors [27].

## Results

Of the 887 subjects who were invited to take part in the main survey, 384 refused by completing and returning a card indicating their unwillingness to take part. A further 91 refused when telephoned or when visited, and 3 gave no response, leaving 409 who were interviewed. It is noticeable that of those who refused, 80% did so by returning the postcard.

Refusal to take part in the main survey varied significantly by age, gender and level of deprivation (Table 1). When fitted into a hierarchical loglinear model each of the three terms showed a significant independent association with refusal. Refusal was higher in the older group, by female gender and increased with increasing level of deprivation. None of the higher order interactions were significant.

**Table 1:** Numbers of subjects refusing and agreeing to take part in the main survey interviews

	**Refused N**	**Interviewed N**	
**Age**			
65–74 years (n = 476)	234 (49.2%)	242 (50.8%)	
75–84 years (n = 412)	244 (59.2%)	168 (40.8%)	χ^2 ^= 8.81, 1df, p < 0.01
**Gender**			
Male (n = 428)	216 (50.5%)	212 (49.5%)	
Female (n = 459)	262 (57.1%)	197(42.9%)	χ^2 ^= 3.90, 1df, p < 0.05
**Deprivation Level**			
Low (n = 351)	165 (47.0%)	186 (53.0%)	
Medium (n = 322)	186 (56.0%)	146 (44.0%)	
High (n = 204)	127 (62.3%)	77 (37.7%)	χ^2 ^= 13.0, 2df, p < 0.001
**TOTAL**	478 (53.9%)	409 (46.1%)	

The 471 people who refused by card return or at personal contact were considered for the follow up study. After GP exclusions a total of 417 were approached. Overall 61% of those who refused to take part in the main survey returned a follow-up questionnaire giving their reasons for refusal. The effect of age, gender and deprivation on the return rate of the follow-up questionnaires was quite different to their effect on participation in the cross-sectional survey (Table 2). Thus the older group had a significantly lower non-participation rate than the younger group. Furthermore, there was little difference between men and women in non-response and the effects of deprivation were inconsistent and non-significant. No higher order interactions were significant.

**Table 2:** Numbers of subjects who replied to the follow-up study

	**Replied N**	**No Reply N**	
**Age**			
65–74 years (n = 205)	116 (56.6%)	89 (43.4%)	
75–84 years (n = 212)	140 (66.0%)	72 (34.0%)	χ^2 ^= 3.93, 1df, p < 0.05
**Gender**			
Male (n = 186)	118 (63.4%)	68 (36.6%)	
Female (n = 231)	138 (59.7%)	93 (40.3%)	χ^2 ^= 0.6, 1df, p = 0.44
**Deprivation Level**			
Low (n = 148)	93 (62.8%)	55 (37.2%)	
Medium (n = 160)	90 (56.3%)	70 (43.7%)	
High (n = 109)	73 (70.0%)	36 (30.0%)	χ^2 ^= 3.35, 2df, p = 0.19
**Total**	256 (61.4%)	161 (38.6%)	

The follow-up questionnaire comprised eight questions, which participants could answer yes, no, or give no response. By far the most commonly selected reason for not taking part in the main study was that the participants considered that they did not do enough exercise to be useful for research (Table 3). Only a minority (28%) indicated that they "were not interested in research" and even these had actually taken part by replying. The other most common answers reflected concerns about loss of privacy, either through research staff visiting them or by being asked questions about personal matters. We examined the data to see if the reasons provided differed between age, gender and deprivation groups (Table 4). Two significant differences were found. Unsurprisingly, older people were more likely to report their age as a reason for non-participation (p < 0.0001). In addition men were more likely than women to indicate that they were not interested in research (33% versus 22%; p = 0.045).

**Table 3:** Reasons for not taking part in the interview study (N = 256)

	Yes (%)	No (%)	No box selected (%)
**A) Misunderstandings about the research**			
I do not do enough activities to be useful for research	55.5	18.1	26.4
I thought the purpose of the study was to encourage people to take part in different activities	32.3	26.0	41.7
**B) Concerns About Privacy**			
I do not want a research nurse coming to my home	44.9	24.0	31.1
I do not want to be asked questions about my personal details/activities	39.4	28.7	31.9
**C) Personal Reasons**			
I am too old to take part in a study	37.4	30.3	32.3
I am not well enough to take part in the study	28.0	39.0	33.1
**D) Not Interested In The Research**			
I am not interested in a study on activities of older people	31.5	33.5	35.0
I am not interested in research	27.4	35.6	37.0

**Table 4:** Reasons for Refusal To Participate By Age, Gender and Deprivation

	Yes (%)	No (%)	No box Selected (%)	χ^2^	p
**A) Misunderstandings about the research**					
***I do not do enough activities to be useful for research***					
65 – 74 years	54	17	28	0.49	0.782
75 – 84 years	57	19	25		
Male	51	23	26	4.00	0.135
Female	59	14	27		
Low deprivation	53	15	32	2.30	0.680
Medium	57	19	24		
High deprivation	56	21	23		
***I thought the purpose of the study was to encourage people to take part in different activities***					
65 – 74 years	31	28	41	0.32	0.852
75 – 84 years	33	25	42		
Male	33	30	37	2.57	0.277
Female	32	23	46		
Low deprivation	33	17	50	6.75	0.150
Medium	30	32	38		
High deprivation	34	30	36		
**B) Concerns About Privacy**					
***I do not want a research nurse coming to my home***					
65 – 74 years	46	22	32	0.30	0.860
75 – 84 years	44	25	30		
Male	45	27	28	1.14	0.567
Female	45	22	33		
Low deprivation					
Medium	41	23	36	1.70	0.792
High deprivation	47	24	29		
***I do not want to be asked questions about my personal details/activities***					
65 – 74 years	40	28	32	0.01	0.995
75 – 84 years	39	29	32		
Male	39	33	28	2.01	0.366
Female	40	25	35		
Low deprivation	35	29	36	2.11	0.715
Medium	39	29	32		
High deprivation	45	27	27		
**C) Personal Reasons**					
***I am too old to take part in a study***					
65 – 74 years	20	41	39	29.10	<0.0001
75 – 84 years	52	21	27		
Male	40	33	28	2.17	0.338
Female	36	28	36		
Low deprivation	38	27	35	0.88	0.927
Medium	38	32	30		
High deprivation	36	33	32		
***I am not well enough to take part in the study***					
65 – 74 years	21	41	38	5.87	0.053
75 – 84 years	34	37	29		
Male	27	43	30	1.59	0.451
Female	29	36	36		
Low deprivation	25	37	38	1.94	0.747
Medium	28	40	32		
High deprivation	32	40	29		
**D) Not Interested In The Research**					
***I am not interested in a study on activities of older people***					
65 – 74 years	35	33	32	1.64	0.441
75 – 84 years	28	34	38		
Male	34	37	29	3.14	0.209
Female	30	30	40		
Low deprivation	28	33	39	2.99	0.559
Medium	33	38	29		
High deprivation	34	29	37		
***I am not interested in research ***					
65 – 74 years	27	36	37	0.02	0.991
75 – 84 years	28	35	37		
Male	33	38	29	6.22	0.045
Female	22	33	45		
Low deprivation	17	33	50	9.39	0.052
Medium	29	38	34		
High deprivation	36	36	29		

Participants were also invited to add their own comments at the end of the questionnaire. Two key themes appeared within the data. The most commonly cited reasons reflected the *perceived difficulty *of participating, rather than a lack of willingness. Many participants indicated that they had too many other commitments to be able to take part in research. These often involved family ties, but could involve other activities:

"My wife does not keep very well and I need to watch over her"

"Family commitments prevent me from attending, organised activities"

"My other commitments will prevent me from participating"

A second dominant theme, *research misunderstanding*, reflected confusion about the nature and purpose of the research itself. Responses suggested that many may have declined to take part in the initial survey *either *because they regarded themselves as already physically active and therefore not appropriate for inclusion, or because they thought that the researchers had contacted them because they were felt to be in need in some way.

"I am pleased as I am, but thank you all for your care and kindness"

"I keep active in my own way, a little gardening etc, when necessary, at my own pace"

"My personal activities at present fulfil my needs"

"I'll no doubt soon be donning the old wooden overcoat"

## Discussion

The study found that for the vast majority of people refusal to participate in the main survey did not reflect an objection to participating in research in principle but frequently stemmed from barriers or misunderstandings about the nature or process of the project itself. The meaningfulness of expressions of non-consent may therefore be called into question.

Response rates for the main survey varied by age, gender and social deprivation. However, this response pattern was different for the follow-up study in which reasons for non-participation were sought. This supports the likely importance of recruitment issues that are research and context specific, as opposed to generic issues alone.

This study is one of a very few that has accessed the views of individuals who have *refused *to take part in a research project. The scale and nature of data collection was constrained by sensitivity to the fact that participants had already refused to take part in our main survey. As a result there are a number of caveats that must be considered prior to discussion of the study findings. Firstly, it is possible that reasons given for non-participation may not reflect those truly responsible. Both social desirability (e.g. unwilling to admit to apathy) and recall may mean some reasons have been omitted. Secondly, the data relates only to older people. While some of the reasons for non-participation may apply to younger groups we do not have the data to support this. Thirdly, it is possible that despite assurances in regard to confidentiality the requirement that the follow-up questionnaire should be sent by their GP may have resulted in some people feeling intimidated and therefore either not responding at all (potentially resulting in some sampling bias) or by responding but providing "acceptable" rather than accurate reasons for original non-participation. Finally, the reasons for non-participation provided are in relation to a request for a detailed, structured interview in the home setting and may not apply to other data collection methods.

Despite these caveats our findings may be useful and informative for researchers in a number of ways. Firstly, they provide detail on likely response rates among older people and the tailoring of information sheets. Secondly, they raise questions over the meaningfulness not only of expressions of non-consent but also consent. Finally, they have implications not only for the design but also the recruitment procedure itself. These are dealt with in turn below.

### Response, refusal and information provision among older people

Fewer than 50% of people invited to take part in a structured interview about social and physical activities agreed to take part. Review papers have reported that many studies among older people have lower response rates [3,6,9,28]. As in this study, others have reported that response rates usually fall with age [6-8] and are lower in those with poorer socio-economic status [9,10]. These variations suggest that the *reasons *for non-participation may be different between social groups and/or that the *strength *of such reasons might differ. Consequently, information sheets or recruitment strategies that are based on a "one size fits all" basis may at best be inefficient and at worst inappropriate.

Within the UK information sheets are increasingly being standardised under COREC (Central Office for Research Ethics Committees) requirements. However, their design is rarely empirically based and almost always atheoretical [14,15], despite the fact that a range of theories of patient behaviour may contain relevant concepts that could be used in their design (e.g. self-efficacy, subjective norm etc) [29]. A Cochrane review of strategies to improve response rates in observational studies revealed a concentration on modes of administration [14,30]. Only two studies focussed on the information leaflets used in recruitment, and these only examined the appropriate level of detail rather than actual content per se. No strategies sought to address the beliefs or attitudes of potential participants towards participation or non-participation. Since this review a number of studies have targeted concerns and demonstrated that recruitment strategies that are designed to address these can have a significant impact [16,31-33]. One recent study demonstrated an increase in participation from 40% to 70% [34].

It is possible that there is so much heterogeneity between trial contexts that established behavioural theory would contribute little as reasons would be trial specific. Indeed despite identifying 15 studies in a Cochrane review of recruitment strategies the authors were uncertain of the generalisability of the finding to other trials as there was too much heterogeneity between studies to perform meta-analysis [15].

### The meaning of non-consent

The information sheet for the main study which had been subject to careful design, consisted of neutral, non-coercive language and had been approved by the local research ethics committee (LREC). However, we found that many expressions of refusal were based on misunderstandings about the research and therefore fell short of being informed and comprehended non-consent.

Ethics committees attempt to ensure that when people *decide to participate *in a research study their consent is meaningful. This depends on three issues: that they are adequately informed, that they adequately comprehend, and finally that they are not coerced. However, LRECs and researchers do not routinely apply the same criteria to *non-participation*. Our data raises questions over the meaningfulness of the expressions of non-consent received in this study. Furthermore, it suggests that even carefully designed and LREC approved information provision can result in poorly comprehended information, undermining meaningful decisions about participation.

There are good reasons why we should seek to ensure that expressions of non-consent (as well as consent) are meaningful and valid. Firstly, it can be argued that potential participants have the right to be provided with full and accurate information prior to making a decision not to participate. Arguably this includes being informed of the consequences of their non-participation (and a low participation rate overall) on the usefulness of the research and for patient care. Just as we should seek to ensure that those who agree to participate do not subsequently regret their decision, so too we should seek to ensure that those who refuse do not look back and wish that they *had *taken part or been given more information. Since many people in our study appeared to have declined participation due to misunderstandings it is quite possible that a correction of these misunderstandings would lead to some regretting their earlier decision.

A second reason to pursue meaningful non-consent stems from the researchers' ethical obligations to those who *do *consent. If we do not actively explore the reasons behind and meaning of non-consent and develop solutions, we may be at risk of wasting the time and generosity of those members of the public who *do *agree to contribute. If consent was given that knowledge would be advanced then researchers must seek to ensure that this is achieved. Obtaining a sufficiently large and unbiased sample may be a prerequisite for this.

Ideally therefore, *refusal *to participate should reflect a fully comprehended and active evaluation of the pros and cons of participation If this is the case, then perhaps non-response or refusal to participate should be regarded as a behaviour that it is not only legitimate to explore and perhaps attempt to change, but one that it would be negligent or unethical not to. However, the challenges of pursuing such research should not be overlooked. The acceptability and ethical implications of studies designed to explore non-participation has been largely omitted from debates around recruitment despite some using arguably intrusive methods. One such study [35] conducted personal visits to the homes of those who had previously written to refuse participation. This approach yielded a final 93% participation rate. Others have only conducted home visits to those who did not respond to an initial request to participate [10,36,37]. Telephone and postal follow-up of all non-responders (including those who refused to participate) have also frequently been used[3,38]. It is interesting that none of these papers mention ethical issues.

### The challenges posed by "opt-in"

We used an "opt-out" approach to recruitment that is increasingly being rejected by research ethics committees in favour of "opt-in" approaches [39]. The vast majority (81%) of those who did not wish to participate in the main survey returned a postcard as part of the opt-out. This would suggest that an active decision against participation had been made. Few studies among older people distinguish between positive opt out and passive non-participation, although two studies involving psychiatric interviews [4,40] also found a high positive opt-out rate. However one of these [40] made up to ten attempts to contact subjects, which may have increased the likelihood that subjects actively opted out. Another study, a trial of influenza vaccination, reported that 50% of those not taking part had also positively opted out [37].

If researchers wish to maintain or increase response rates to research and ensure that consent and non-consent is informed and meaningful then an understanding of the issues that concern potential participants is crucial [1], as is a knowledge of concepts that are known to influence behaviour more generally [41]. However, the increasing requirement to use opt-in procedures may undermine such attempts. Under the opt-out procedure recruitment depends largely on the inertia of individuals [39]. However, the move towards an "opt-in" system has now reversed this. The failure to act leads to non-inclusion, but also means that non-inclusion is less likely to stem from any meaningful consideration of the pros and cons. Consequently, "non-participation" may not be an intentional or volitional act reflecting an unwillingness to take part but rather reflect a lack of intention or volition altogether, particularly among individuals who are less inclined to be actively involved in health-related decision making but instead leave it to professionals. Indeed a recent randomised trial has found evidence to support this [2]. Non-participation may therefore sometimes stem less from a cognitive process and more from an adopted and established role in relation to health services generally. Furthermore, if data suggests that some individuals may have a preference *not *to be involved in *some *decisions, this might support Parker's recent argument for the use of data without consent for some low-risk research: "Patients may have good reasons to expect, or come to expect, that their records will be used without their consent for some low risk research, under certain conditions. Where this is the case, such expectations provide reasonable grounds for considering such research to be ethical."[42]p183.

## Conclusion

Findings from this study have potential implications for recruitment and consenting practices when seen against the context of recent changes to ethical requirements and data protection. Firstly, the design of information leaflets and the verbal consenting approach should not be excluded from the concept of evidence-based practice. If there is empirical evidence or relevant behavioural theory to suggest the best means to achieve meaningful consent then these should be pursued. Secondly, expressions of non-consent need to be subject to further research and not seen as out of bounds as a focus for investigation. A limited amount of additional research may be important to ensure we are conducting our research recruitment to a high standard.

Thirdly, in pursuing the ideal of meaningful consent researchers could consider presenting potential participants with two options: participation and non-participation and highlight the pros and cons of each in a manner not dissimilar to that of two treatment options. At present, it could be argued that the information insisted upon by ethics committees focuses on that needed to take part rather than that needed to decide *not *to take part. The consequences of non-participation for example (for both the individual, science, and the public), are rarely conveyed, probably through fear of being coercive. While recent commentators have questioned whether we currently know what information potential participants need in order to participate in a research project it may also be worth considering what information they may require in order to know whether they should *refuse *to participate [1]. The presentation of such information may contribute to ensuring that *both *consent and non-consent are meaningful.

## Competing interests

The author(s) declare that they have no competing interests.

## Authors' contributions

IKC and LI conceived the idea for the study. All authors were involved in the design. AM collected the data. LI and IKC analysed the data. BW wrote the paper. All authors edited, revised and approved the final manuscript.

## Pre-publication history

The pre-publication history for this paper can be accessed here:


